# Editorial: Gut microbiome, metabolites, and immune response in lupus, rheumatoid arthritis, and Sjögren syndrome

**DOI:** 10.3389/fimmu.2022.1126800

**Published:** 2023-01-16

**Authors:** Lidan Zhao, Aleksandar David Kostic, Xuan Zhang

**Affiliations:** ^1^ Department of Rheumatology and Clinical Immunology, National Clinical Research Center for Dermatologic and Immunologic Diseases (NCRC-DID), Peking Union Medical College Hospital, Chinese Academy of Medical Sciences, Beijing, China; ^2^ Section on Islet Cell and Regenerative Biology, Joslin Diabetes Center, Boston, MA, United States; ^3^ Department of Microbiology, Harvard Medical School, Boston, MA, United States; ^4^ Department of Rheumatology, Beijing Hospital, National Center of Gerontology, Institute of Geriatric Medicine, Chinese Academy of Medical Sciences, Beijing, China

**Keywords:** immunometabolism, mitochondrion, gut microbiome, systemic lupus erythematosus, Sjogren syndrome

The dynamic homeostasis of the human immune system and the functional polarization of immune cells are fine -tuned by intracellular metabolic processes and energy supplies. Immune cells reprogram their metabolic predilection to adapt to their bioactivities and energy requirements. Gut microbiota, on the other end, influence nutrient absorption and transformation, impact immune tolerance and competence *via* metabolites like short-chain fatty acids, and link to the metabolic reprogramming of immune cells directly or indirectly.

The gut microbiota status coordinates with the host immunometabolism profile to determine the activation, proliferation, and differentiation of immune cells. Therefore, both immunometabolism machinery and gut microbiota may provide promising targets for curbing unwanted inflammation and autoreactivity in autoimmune diseases.

In this recent collection, the association of core metabolic pathways —including glycolysis, lipid metabolism, and mitochondrial function— with the initiation and perpetuation of inflammation and autoimmunity was comprehensively reviewed, and the role of gut microbiota and their metabolites in typical autoimmune diseases like systemic lupus erythematosus (SLE), Sjögren syndrome (SS) and rheumatoid arthritis (RA) were explored and their participation in immunometabolism discussed. 

Glucose and lipids act more than energy resources, they are also immune mediators and signaling molecules that are deeply involved in the immune response direction. Dyslipidemia in SLE has been reported to contribute to increased immature cardiovascular disease and is suggested to be associated with the pathogenesis of lupus. Sun et al. comprehensively expounded the lipid metabolism, lipid rafts changes, and their involvement in immune cells’ functional modulations. Cholesterol efflux interferes with the assembly of lipid rafts and influences the threshold of T-cell receptor activation and B-cell receptor signaling. Lipid hydroperoxides, lipid synthesis and degradation, and correlated regulators and metabolites participate widely in immune cell activation and cytokine production. Signaling interventions of lipid metabolism, supplementation of beneficial metabolites like omega-3 fatty acids and short-chain fatty acids might be hopeful adjuvant therapy in SLE.

Glycolysis is a low efficient but fast way to acquire energy, which is the preference of cells in rapid growth and proliferation. Xu et al. specifically summarized the role of glycolysis in the differentiation and functional polarization of innate immune cells and discussed the possible mechanisms of glycolysis in the pathophysiological processes of autoimmune diseases. Several anti-glycolysis drugs have been mentioned as promising therapies for autoimmune diseases, among which rapamycin and metformin have acquired wide attention and been researched in certain clinical conditions.

Oxidative phosphorylation in mitochondria is a highly efficient way of generating adenosine triphosphate with the byproduct of reactive oxygen species (ROS). Zhao et al. gave an in- depth review of the distinctive mitochondrial functions and metabolic pathways involved in adaptive and innate immune cell function. By illustrating the excessive production and defective clearance of pathogenic mitochondrial ROS, and the mechanism of immunogenic mitochondrial DNA participating in the immunopathogenesis of lupus, they explicated the potentiality of mitochondria intervention in lupus treatment.

Consistent with previous studies, Toumi et al., confirmed the existence of gut dysbiosis in SLE patients with reduced gut microbiota diversity and lower Firmicutes/Bacteroidetes (F/B) ratio *via* 16S rRNA sequencing. What is more, they also identified a core set of 6 taxa with differential abundance in patients with active and inactive diseases. SLE patients and pristane-induced lupus mice shared some alterations of gut microbes phenotype, and the cross-species common changes highlight the importance of gut microbiota in the development of SLE.

*Lactobacillus* is considered beneficial for humans and widely used in producing fermented foods like yogurt. Different species may function differently on immune cells, thus influencing lupus progression in different ways. For example, *Lactobacillus reuteri* has been verified to be pathogenic in SLE *via* promoting type 1 interferon production. Wang et al. summarized the two sides of *Lactobacillus* in the occurrence, development, treatment, and prognosis of SLE, and discussed the potential immunological effects of different strains of *Lactobacillus* on lupus.

As the most complicated and heterogenous autoimmune diseases that can involve almost all body parts and currently have limited remedies, SLE remains the most formidable and challenging disease for rheumatologists. Jiang et al. reported the efficacy of a new therapeutic strategy of whole blood exchange (WBE) in refractory severe hematolytic anemia secondary to SLE. Going further than plasma exchange, WBE could theoretically remove the pathogenic substances in the blood more thoroughly, regardless of whether they are soluble or cell-binding. Indeed, it seemed in the small cohort WBE showed a rapid effect with the advantages of less glucocorticoid consumption and shorter hospital stay, although these advantages seemed to fade one month later. Still, this exploratory treatment could be a promising strategy for those severe patients who are irresponsive to current therapy.

Sjögren’s syndrome (SS) also attract scientists’ interests regarding its associated gut dysbiosis. Yang et al. reported their findings of 16S rRNA gene sequencing of fecal samples and metabolome data from SS patients. Increased abundances of proinflammatory microbes and decreased abundances of anti-inflammatory microbes were found in these patients and enriched metabolites were suggested to be involved in amino acid metabolism and lipid metabolism. By using correlation analysis, the authors also reported the possible associations of pathogenic microbes like *Escherichia-Shigella* with some of the enriched metabolites. Meanwhile, Deng et al. summarized the current observations of microbial shifts reported in the gut and the oral microbiome of SS patients, which may be associated with disease activity and proinflammation factors levels. The altered microbiome may activate the immune system *via* molecular mimicry, metabolite changes, and epithelial tolerance breakdown, then result in chronic inflammation and damage in exocrine glands, particularly the salivary and lacrimal glands. The proposed gut-ocular and gut-oral axis remain to be explored in causality studies and pathogenic dissection research.

Focusing on the differentiation and function of megakaryocytes and platelets, Durholz et al. showed the supplementation of propionate in collagen- induced arthritis (CIA) mouse model can alleviate arthritis, which was accompanied by a decreased but more matured megakaryocyte erythrocyte progenitor population in bone marrow. The platelet coagulation reactivity to adenosine diphosphate induction was selectively increased. Also, the author carried out RNAseq and subsequent KEGG analysis and declared propionate could upregulate megakaryocyte differentiation and platelet activation pathways. Increased acetylation and propionylation for histone 3 in megakaryocytes after incubation with propionate were detected. Though brief and preliminary, this research is interesting and provides a new visual angle for looking into the function of short-chain fatty acids.

## Author contributions

All authors listed have made a substantial, direct, and intellectual contribution to the work and approved it for publication.

